# Association Between Vitamin D and Minor Salivary Gland Inflammation

**DOI:** 10.7759/cureus.32160

**Published:** 2022-12-03

**Authors:** Vivek Mehta, Ruben Peredo-Wende

**Affiliations:** 1 Department of Rheumatology, Alaska Native Tribal Health Consortium, Anchorage, USA; 2 Department of Rheumatology, Albany Medical Center, Albany, USA

**Keywords:** sjogren and sicca, vitamin-d, 25 (oh) vitamin d, primary sjogren’s syndrome, rheumatology & autoimmune diseases

## Abstract

Objective

There has been increasing research regarding the effects of vitamin D on autoimmune disorders. There is some evidence of a correlation between vitamin D levels and sicca symptoms. We attempted to evaluate the correlation between vitamin D levels and inflammation of the minor salivary gland (MSG).

Methods

Data for 214 patients who had undergone MSG biopsy were reviewed. Eighteen patients with other autoimmune/neoplastic processes were excluded. Seventy-seven of 196 patients had serum Vitamin D levels available and were selected for this retrospective study. Demographic, clinical, and immunological features, extra-glandular manifestations, autoantibodies, and clinical laboratory tests were collected and compared between patient groups with a focus score (FS) of 0 and 1 or more.

Results

Out of 77, 29 patients had an FS of 0 while 48 had an FS of 1 or more. Mean vitamin D levels were noted to be lower in patients with an FS of 1 when compared to patients with an FS of 1 or more.

Conclusion

In this study, patients with an FS of 1 were noted to have low vitamin D levels but this association was not seen at a higher FS.

## Introduction

Sjogren’s syndrome (SS) is a chronic autoimmune inflammatory disease targeting the exocrine glands. The resultant gland dysfunction is most commonly manifested by mucosal surface dryness, causing dryness in the eyes and mouth after years of relentless disease progression [1,2]. Subsequently, the diagnostic peak age falls in middle-aged individuals and increases in the elderly population [3]. Like many other autoimmune diseases, SS has a predilection for the female gender, with an estimated ratio of 9:1 [4]. While SS presenting alone is labeled as primary SS (pSS), it is named secondary Sjogren’s syndrome (sSS) when presenting with other another autoimmune disease [5]. SS has protean disease manifestations and while most pSS presents with xerostomia, xerophthalmia, and fatigue, the extra-glandular manifestations may have various degrees of severity and organ involvement. In the most severe cases, pSS can be associated with vasculitis, cryoglobulinemia, and mucosal-associated, lymphoid tissue-related lymphoma.

The precise mechanisms of the immune disruption with subsequent loss of tolerance against the exocrine glands are not fully clear. Similar to many other autoimmune diseases, genetically susceptible individuals are prone to develop the disease if exposed to one or many environmental hazards. Susceptibility was identified in the presence of Class II HLA-DR and HLA-DQ alleles in multiple ethnic backgrounds and additionally Class I HLA-B8 in Caucasians. Viruses like herpes virus 6, cytomegalovirus, Epstein-Barr virus, hepatitis C virus, etc. have been postulated to play a role in the etiopathogenesis and perpetuation of SS, supported by the disarray of the interferon imprint highly expressed in this disease, similar to what is seen in systemic lupus erythematosus (SLE) [6-8].

Sjogren's syndrome is known to cause salivary gland inflammation. A minor salivary gland (MSG) biopsy is often undertaken to help evaluate for salivary gland inflammation and support diagnostic suspicion when clinical and laboratory data are insufficient. A focus score of 1 or more is usually indicative of MSG focal lymphocytic sialadenitis. The focus score is the resultant of the measurement between the number of focal lymphocytic infiltrates, or focal lymphocytic sialadenitis adjacent to intact acini or ducts, over the 4 mm^2^ of salivary gland tissue. Readings are best obtained with at least 12-15 mm^2^ of gland specimen obtained from a lip biopsy. Focal lymphocytic sialadenitis is the accumulation of 50 or more lymphocytic cells with few plasmocytic cells [9]

Vitamin D has been shown to decrease pro-inflammatory cytokines and increase anti-inflammatory cytokines in in-vitro studies [10]. The role of vitamin D in autoimmune disorders has been an area of interest recently with some ecologic observations suggesting higher prevalence at northern latitudes [11]. Vitamin D has been shown to have possible adverse effects on dry eye symptoms and some studies have suggested an association between vitamin D levels and SS [12,13]. Minor salivary gland (MSG) biopsy is an important diagnostic tool in SS and has been shown to have prognostic value [14,15]. Considering the role of vitamin D in cytokine regulation and immunity, we undertook this study to evaluate for correlation between inflammation in MSG and serum vitamin D levels.

## Materials and methods

A review of 214 patients who had an MSG biopsy was undertaken at a tertiary care center. Eighteen patients with autoimmune disorders other than Sjogren syndrome or with neoplastic processes were excluded. Pathology results of the remaining 196 patients were reviewed. Chemiluminescence immunoassay results for serum VD levels (25-100 ng/mL) were available in 77 patients. 

Serum vitamin D levels were assessed in the context of the severity of sialadenitis, as well as other clinical, laboratory, and serological data. Demographic, clinical, and immunological data were collected, including sicca symptoms, extra-glandular manifestations, autoantibodies, and clinical laboratory tests. The clinical data were compared between patients with a focus score of 0, and 1 or more. The protocol was approved by the institutional review board (IRB) of Albany Medical Center.

The definition of Sjogren’s syndrome was based on the 2002 American-European Classification criteria for Sjogren’s syndrome. In it, criteria for SS were established based on a combination of a composite of six possibilities. Objective criteria comprised the positive histopathology for focus scoring of ≥1; positive serology of either anti-SSA/Ro or anti-SSB/La antibodies; ophthalmologic evidence of dry conjunctivae (≥4 van Bijsterveld’s scoring of ocular dryness) or abnormal Schirmer’s test without anesthesia (≤5 mm in 5 minutes); and objective evidence of poor saliva production ≤ 1.5mL of unstimulated saliva in 15 minutes, or parotid sialography with the presence of diffuse sialectasis and abnormal salivary scintigraphy showing delayed uptake, reduced concentration and/or delayed excretion of tracer. Subjective symptoms were the set of dry eye sensations over three months, sand or gravel sensation in eyes, and the need to use tear substitutes more than three times per day. Oral dryness comprised mouth dryness sensation for more than three months, a history of swollen glands, and the need to refresh the mouth to swallow foods. If the case met three or more objective criteria, or 4 of a combination of objective and subjective criteria, with the presence of either abnormal serology or histopathology, the definition criteria for SS were met.

Statistical analysis included the student's t-test and Mann-Whitney U test with results indicated as mean ± standard error of the mean (SEM) and parametric and nonparametric correlations. A two-tailed value of p<0.05 was taken to indicate statistical significance.

## Results

The study population consisted of 77 patients who had vitamin D levels available. Out of 77 patients, 29 had an FS of 0 and 48 had an FS of 1 or more. The demographics of the patients with an FS of 0 and 1 or more are described in Table 1.

**Table 1:** Demographics of patients with an FS of 0 and an FS of 1 or more FS: focus score

	FS=0 (N=29)	FS ≥ 1 (N=48)
Age (Mean +/- SD)	49.1 +/- 9.8	51.5 +/- 11.4
Female	26 (89.6%)	40 (83.3%)
Male	3 (10.3%)	8 (16.6%)

We collected data regarding patients’ SS-related clinical features and laboratory results, including the serological markers commonly associated with SS, common extra-glandular manifestations, and comorbidities. Vitamin D levels were compared in patients with symptoms and patients without symptoms in both groups of people, with an FS of 0 and an FS of 1 or more. In patients with an FS of 1 or more, vitamin D was noted to be lower in those with comorbidities of hypothyroidism (mean of 19.5 vs 31, p=0.022) and fibromyalgia (mean of 22.2 vs 32.7, p=0.016). Vitamin D also correlated with a high erythrocyte sedimentation rate (ESR) (mean 10.8 vs 26.8, p= 0.048) (Table 2).

**Table 2:** Vitamin D correlation with various parameters in people with an FS of 0 and an FS of 1 or more FS: focus score; SS: Sjogren’s syndrome; ILD: interstitial lung disease; CRP: C-reactive protein; ANA: antinuclear antibody; ESR: erythrocyte sedimentation rate; IgG: immunoglobulin G

	FS = 0 (N=29)			FS ≥ 1 (N=48)		
	N (%)	Vitamin D levels (ng/ml)		N (%)	Vitamin D levels (ng/ml)	
		Positive/ Abnormal	Negative/ Normal		Positive/ Abnormal	Negative/ Normal
SS-related clinical features						
Dry eyes	21 (72.4)	28.4 +/- 14.8	26.1 +/- 8.1	35 (72.9)	26.8 +/-13.1	33.2 +/- 19
Dry mouth	19 (65.5)	26 +/- 14.7	31.2 +/- 9.7	39 (81.3)	27.7 +/-14.7	32.2 +/- 16.8
Schirmer’s test	12/26 (46.2)	30.3 +/- 12	25.9 +/- 13.8	23/34 (67.6)	28.1 +/- 14.7	23 +/- 10.6
Extra-glandular manifestations/comorbidities						
Fatigue	27 (93.1)	26.9 +/- 13.4	40.5 +/- 7.8	47 (97.9)	28.8 +/- 15.1	16
Arthritis	7 (24.1)	28.9 +/- 14.9	27.5 +/- 13	8 (16.7)	33.8 +/-22.2	27.4 +/- 13.3
Raynauds	6 (20.7)	34.8 +/- 15.1	26 +/- 12.4	13 (27.1)	27.4 +/- 13.9	29 +/- 15.6
ILD	0	N/A	27.8 +/- 13.2	6 (12.5)	37.7 +/- 25.3	27.2 +/- 12.9
Fibromyalgia	10 (34.5)	27.6 +/- 14.3	27.9 +/- 13	19 (39.6)	22.4 +/- 13.4	32.7 +/-14.7
Hypothyroidism	1 (3.5)	13	28 +/- 28.32	11 (22.9)	19.5 +/- 9.8	31.2 +/- 15.3
Serology						
ANA	9 (31)	25.1 +/- 12.1	29 +/- 13.8	23 (48.9)	31.2 +/- 16.8	26.5 +/- 13
Anti-SSA/Ro	0	N/A	27.8 +/-13.2	13/26 (28.3)	31 +/- 21	27.6 +/-12.7
Anti-SSB/La	0	N/A	27.8 +/- 13.2	4/46 (8.7)	38.3 +/- 18.5	27.6 +/- 14.9
Rheumatoid factor, IU/ml	3 (10.3)	18.3 +/- 13.7	28.9 +/- 13	7/45 (15.6)	28.1 +/- 12.7	27.8 +/-15
Laboratories						
Lymph. count = 1.4 x cells/hpf	8 (27.6)	29.8 +/- 13.8	27 +/- 13.3	14/42 (33.3)	33.1 +/- 18.6	27.6 +/- 12.2
CRP > 0.8 mg/dl	3/28 (10.7)	34 +/- 13.5	27.6 +/- 13.3	14/44 (31.8)	26.9 +/- 19.3	27.8 +/- 12
ESR > 20 mm/hr	4/28 (14.3)	18.8 +/- 10.9	29.9 +/- 13.1	10/45 (22.2)	25. 4 +/- 20.1	28.4 +/- 12.6
IgG > 1520 mg/dl	1/19 (5.3)	52	24.6 +/- 12.9	6/35 (17.1)	23 +/- 17.9	26.3 +/- 11.7

Vitamin D levels were comparable in patients with an FS of 0 and 1 or more (mean of 28.5 vs 27.8, p=0.825). We further attempted to evaluate if there was any measurable difference in vitamin D levels depending on the severity of sialadenitis by comparing patients with an FS of 0, 1, and > 1. The population with an FS of 1 was noted to have vitamin D levels lower than patients with an FS of more than 1 (p=0.022) (Table 3).

**Table 3:** Focus score and vitamin D levels in patients who underwent an MSG biopsy FS: focus score; MSG: minor salivary gland

	FS<1	FS=1	FS>1
N (%)	29 (37.6%)	12 (15.6%)	36 (46.7%)
Vitamin D levels (mean +/- SD)	27.7 +/- 13.2	19.9 +/- 14.3	31.4 +/- 14.3

## Discussion

Most autoimmune disorders are thought to have genetic and environmental components that contribute to their pathogenesis. There has been increasing interest regarding the role of vitamin D as an environmental factor affecting autoimmune disorders. Vitamin D has been shown to have effects on both innate and adaptive immunity [16]. Correlation has been noted between north to south latitude, sun exposure, vitamin D levels, and autoimmune disorders like rheumatoid arthritis, multiple sclerosis, systemic lupus erythematosus, and inflammatory bowel disease [17-19].

SS is an autoimmune disorder affecting the exocrine glands, most commonly attacking the salivary and lacrimal glands, which results in sicca symptoms. Low vitamin D levels have been documented in patients with pSS and have been found to have an inverse correlation with pSS [20,21]. Lee et al. recently described a correlation between vitamin D levels and the severity of dry eye symptoms.

Our study was undertaken to evaluate for correlation between vitamin D levels and salivary gland inflammation. Salivary gland inflammation is usually evaluated by an MSG biopsy, quantified in FS. FS refers to the number of foci of lymphoid tissue per surface area of 4 mm², with each focus defined as a collection of 50 or more mononuclear lymphoid cells [22,23]. The severity of sialadenitis is described on a scale of FS 0 to 4 [24].

Our data suggested that while the vitamin D level was noted to be lower in patients with an FS of 1 when compared to patients with an FS of 0, no such difference was noted at higher FS. It is possible that the effects of vitamin D were most prominent in patients with a low level of sialadenitis and disappeared at a more advanced level of inflammation.

There were fewer patients with FS 1 compared to other groups, which may affect the measured outcome. Due to the retrospective nature of this study, it is also prone to a selection bias. Most patients with clinically apparent SS would not undergo an MSG biopsy, increasing the chances of atypical causes of MSG inflammation. Occasionally, patients with nonspecific inflammation can be mistakenly applied an FS [25]. Another possible pitfall of FS overestimation is due to limited tissue availability. Therefore, some evidence of discordance between FS and sicca symptoms, and similarly the effects of vitamin D and sialadenitis might be more complicated than one would imagine [9].

Prospective studies might be a better model to explore a possible dynamic interplay between FS and vitamin D levels. However, evidence reveals that the effects of factors like vitamin D on FS might be complex to study. According to a study looking at MSG inflammation over the years, the quantity of inflammatory infiltrating cells and cell types in patients with Sjogren’s syndrome does not seem to change over the years [26]. It appears that the number and type of infiltrating mononuclear lymphocytic cells might be an early event, remaining stable in time and despite the subsequent exposure to multiple inflammatory stimulators. In an animal model study with normal lacrimal glands aimed at identifying precursor of Sjogren foci in histologically normal glands, the authors identified a cluster of transcripts associated with Sjogren’s foci composed of several mRNAs, chemokines, and cytokines (with prevailing BAFF), suggesting many molecular changes that occur before the histologic findings of FS can be detected [27]. Analog to this study, perhaps vitamin D measurement early in the disease during FS formation might capture a more meaningful correlation with low vitamin D levels in those carriers of this molecular signature if one is present.

## Conclusions

Our study showed lower vitamin D levels in patients with an FS of 1, but this correlation was not seen at higher FS levels. Considering the limitations of this retrospective study, further prospective controlled studies are needed to confirm this association. It is possible that vitamin D levels influence the early phase of MSG inflammation and may not be as relevant once the disease state reaches a higher FS. The role of vitamin D in people with Sjogren's syndrome remains controversial.
